# Toward an Improved Understanding of the Ionizing Radiation Induced DNA Damage/Response Networks in Human Malignancies

**DOI:** 10.3389/fonc.2014.00335

**Published:** 2014-12-04

**Authors:** Daphne A. Haas-Kogan, David R. Raleigh, Adam Paul Dicker

**Affiliations:** ^1^Departments of Radiation Oncology and Neurological Surgery, Helen Diller Family Comprehensive Cancer Center, UCSF Benioff Children’s Hospital, University of California San Francisco, San Francisco, CA, USA; ^2^Department of Radiation Oncology, University of California San Francisco, San Francisco, CA, USA; ^3^Department of Radiation Oncology, Kimmel Cancer Center, Jefferson Medical College, Thomas Jefferson University, Philadelphia, PA, USA

**Keywords:** ionizing radiation, ionizing radiation induced DNA damage, DNA damage, radiation, editorial

## Glioblastoma EGFR Mutation Predicts the Response to Cytotoxic and Targeted Therapies

Several molecular aberrations in glioblastoma have been shown to have prognostic significance, and investigations of targeted therapies for glioma patients are ongoing. Here, Wachsberger and colleagues demonstrate that overexpression of a constitutively active form of the epidermal growth factor receptor (EGFR) found in some glioblastomas sensitizes cells to both multimodal cytotoxic therapy and cetuximab (1). The data further indicate that EGFR mutation may predispose gliomas to defective homologous recombination repair (HRR). These findings are relevant to future clinical trials, and suggest that targeted agents may achieve the greatest benefit in glioma patients who have been stratified according to molecular markers.

## MicroRNA Mediates the Cellular Response to DNA Damage

DNA damage triggers a signaling cascade that affects expression, subcellular localization, and activity of mediators; the strength and nature of molecular interactions; and chromatin structure. Ongoing discovery of novel effectors has amplified understanding of the complex network that mediates the DNA damage response (DDR). Recent evidence demonstrates that genotoxic stress regulates expression of distinct microRNAs (miRNA), some of which have been shown to directly influence expression of DDR effectors including ATM, DNA-PKcs, and BRCA1. The links between miRNAs and the DDR are summarized here by Wright and colleagues, as are the implications of future investigation into this emerging field (2).

## Modulation of Double-Strand Break Repair Pathways to Radiosensitize Cancer Cells

Disparate sensitivity to the cytotoxic effects of radiation mediates selective tumor cell killing with relative preservation of normal tissues. Significant effort has been dedicated to understanding the mechanisms underlying differential radiosensitivity with the ultimate goal of enhancing the therapeutic ratio of ionizing radiation. Homologous recombinational repair is among the primary pathways utilized by cells to process otherwise lethal DNA damage, and is therefore a promising target to improve the efficacy of radiation therapy. The major pathways of double-strand break repair are summarized here by Mladenov and colleagues, as are potential targets for radiosensitizing cancer cells (3).

## Radiation-Induced Second Neoplasms

Every year, more than 700 childhood cancer survivors will join the 300,000 individuals in the United States who have survived their cancer diagnoses, but will struggle with the consequences of the treatments that cured them. Twenty-five years after the first cancer diagnosis, the death rate due to subsequent malignancies exceeds that due to all other causes. Technological advances have transformed the delivery of radiation, resulting in more conformal radiation plans. We must investigate whether the benefit of decreasing acute side effects comes at a cost of lower-dose radiation distributed throughout the child’s body that may increase the risk of fatal radiation-induced cancers. Braunstein and colleagues highlight such investigations into the current state of epidemiologic modeling and radiotherapy delivery data and their impacts on patient care (4).

## The Combination of Novel Targeted Molecular Agents and Radiation in the Treatment of Pediatric Gliomas

A comprehensive, cutting edge approach to gliomas requires first a critical understanding of the molecular underpinnings of glioma pathogenesis and response to therapy. Advances in molecular, genetic, and biochemical technologies have provided unprecedented insight into glioma biology and these are reviewed herein by Dasgupta and colleagues (5). Targeted therapeutics are available for many implicated pathways; however, follow up biological studies defining the appropriate, most effective agents for each alteration are lacking. Clinical studies must be designed that collect and harness molecular data within prospective trials, in order to generate pre-clinical and clinical data that will determine the best combination of agents for each molecular subtype.

## Ionizing Radiation in Glioblastoma Initiating Cells

Rivera and colleagues explore the mechanisms underlying radiation resistance in GBM initiating cells (GICs) that are critical to enhanced therapeutic efficacy against neural progenitor cells (NPCs) (6) The importance of this approach is highlighted by several recent reports of improved glioma control and clinical outcome when aggressive therapy and higher doses of radiation are administered to the adult human subventricular zone, which constitutes the largest area of neurogenesis and houses the greatest concentration of NPCs. Such approaches are now possible with our modern arsenal of sophisticated dose delivery technology, with which we can modulate the dose to substructures of the brain.

## Hypofractionated Radiation and Mouse Models of Non-Small Cell Lung Cancer

Recent clinical trials have demonstrated high rates of long-term local control and survival from early stage non-small cell lung cancer (NSCLC) treated with stereotactic body radiotherapy (SBRT). Compared to conventionally fractionated radiation regimens, the success of SBRT for NSCLC suggests that hypofractionated irradiation may induce distinct molecular pathways to enhance cell killing. Using two genetically engineered mouse models of NSCLC, Perez and colleagues demonstrate superior tumor control with hypofractionated radiation. The cellular response to SBRT is incompletely understood, but the genetic reagents described here will be useful to elucidating the molecular mechanisms that underlie the SBRT DDR (7).

## Conflict of Interest Statement

The authors declare that the research was conducted in the absence of any commercial or financial relationships that could be construed as a potential conflict of interest.
